# Predictors of Hypertension among Nonpregnant Females Attending Health Promotion Clinic with Special Emphasis on Smokeless Tobacco: A Cross-Sectional Study

**DOI:** 10.1155/2017/8765217

**Published:** 2017-08-16

**Authors:** Devika Bhatt, Shashi Sharma, Ruchika Gupta, Dhirendra N. Sinha, Ravi Mehrotra

**Affiliations:** National Institute of Cancer Prevention and Research (NICPR), Noida, India

## Abstract

**Aim:**

To determine the predictors of hypertension among nonpregnant females attending a health promotion clinic.

**Design and Setting:**

A cross-sectional study was conducted during March to June 2016, at the National Institute of Cancer Prevention and Research, India.

**Methods:**

The study included 319 nonpregnant females of age 20–70 years. Demographics such as age, literacy, and income were noted. History regarding use, frequency, and quantity of smokeless tobacco was taken. Height, weight, and blood pressure were measured and body mass index was calculated.

**Statistical Analysis:**

Pearson's product-moment correlation coefficient was calculated between each of the variables of age, smokeless tobacco consumption, and body mass index versus systolic and diastolic blood pressure, respectively. The linear as well as multiple linear regression analysis was employed to identify the risk factors for hypertension.

**Results:**

A univariate linear regression analysis showed that age, smokeless tobacco consumption, and body mass index were associated with systolic blood pressure (*P* value < 0.001 for each). For diastolic blood pressure, high body mass index was a predictor. Multiple linear regression analysis showed that both systolic and diastolic hypertension were associated with high body mass index and low level of education. Moreover, the systolic hypertension was associated with higher age and smokeless tobacco use.

**Conclusion:**

Health promotion requires control of body mass index and smokeless tobacco cessation for preventing hypertension and its complications.

## 1. Introduction

Hypertension (HTN) is an extremely common disease cutting across the territorial boundaries of the countries. HTN has been labeled as a “silent killer” due to the associated risk of ischemic heart disease and cerebrovascular accidents [1]. To curb the menace of hypertension, the Indian government launched the National Program for Diabetes, Cardiovascular Disease, and Stroke in 2008. Cancer prevention was further integrated into this program to call it as the national program for prevention and control of cancer, diabetes, cardiovascular disease, and stroke. Identification of risk factors for HTN and control of blood pressure are among the major interventions envisaged in the program [2]. Age, race, and family history are some of the nonmodifiable risk factors for HTN. More important are the modifiable factors like body weight, the level of physical activity, the sodium content of diet, diabetes, and consumption of tobacco and alcohol [3]. Of these, overweight or obesity has been documented as an important predictor of HTN in western as well as Indian studies [4, 5]. Though association of smoking with HTN is well understood [6, 7], the relationship of smokeless tobacco (SLT) with HTN is still not very clear [8, 9]. Earlier studies from India have shown a significant increase in the prevalence of HTN among SLT users [10, 11]. The Global Adult Tobacco Survey (GATS) from India during 2009-10 showed that 24% of males and 17% of females use SLT products and this proportion is higher compared to smoking in both genders (15% of males and 2% in females) [12]. Most of the earlier Indian studies exploring the association of HTN with SLT use have included predominantly male subjects in their study population. In a country like India with a high prevalence of SLT use among females, evaluation of the effect of SLT use on HTN in females is imperative. The present study, conducted at a health promotion clinic, was aimed at evaluating predictors for HTN among nonpregnant females with special emphasis on smokeless tobacco.

## 2. Materials and Methods

All adults visiting our health promotion clinic during the four-month period from March till June 2016 (*n* = 511) were screened for cervical cancer and breast cancer (in females), oral cancer, diabetes mellitus, hypertension, and obesity. Of these, 319 nonpregnant females in the age range of 20–70 years who had blood pressure measurement and gave informed consent to participate in the study were included. Pregnant females and women using smoked forms of tobacco (cigarettes, bidis, or hukka) were excluded from the study. These patients were excluded due to their low numbers, the possible confounding by pregnancy-associated hypertension and changes in body mass index (BMI) during pregnancy. The small numbers of males screened in the clinic could possibly result in bias of the results of the study and hence were excluded. The sample size was calculated by assuming the sample correlation of 0.35 which has come from the population with a correlation of 0.50, *α* (type 1 error) of 5%, and power of the study to be 90%. A total of 314 participants were required to be enrolled in the study. The quantity of smokeless tobacco was derived by taking the product of packets per day and years of consumption of SLT. For all the participants, demographic parameters including age, education, and family income were noted. Height and weight were recorded and the BMI was measured using Omron HBF-212-IN (Omron Healthcare Ltd., Kyoto, Japan). Blood pressure was measured in sitting position after 5 minutes of rest, in the left arm by using Omron JPN 1 (Omron Healthcare Co. Ltd, Kyoto, Japan). Participants were labeled as hypertensive as per the definition of Joint National Commission-8. The JNC-8 defines hypertension as the systolic blood pressure more than or equal to 140 mm of Hg and diastolic blood pressure as more than or equal to 90 mm of Hg on two or more properly measured blood pressure readings from two or more clinical visits, among adults 18 years and older. The blood pressure goals for adults more than sixty years old are <150/90 [3].

Statistical analysis was done by using IBM SPSS, version 20. A descriptive analysis was done for quantitative data and a multiple linear regression analysis was done for predicting the risk factors of blood pressure. Descriptive statistics was expressed as the mean ± standard deviation (SD). Two-sided *P* value< 0.05 was the significance level.

## 3. Results

The age range of participants was 20–72 years with mean ± SD being 38.62 ± 10.04. Approximately half (45%) of the study participants were illiterate, 9.7% had a primary education (grades I–V), 12.7% had an upper primary education (grades VI–VIII), 23.2% had a secondary education (grades IX–XII), 7.2% had a graduate degree, and only 1.6% had a postgraduate degree. The majority (67.4%) of the study participants had a monthly income between 5,000 and 10,000 INR per month while 13.8% had a monthly income below 5000 INR per month, 16% had a monthly income between 10,000 and 30,000 INR per month, and only 2.8% had a monthly income above 30,000 INR per month.

The systolic blood pressure ranged from 92 to 206 mm Hg with the mean ± SD being 127.13 ± 19.72. The diastolic blood pressure ranged from 55 to 165 mm Hg, mean ± SD being 80.39 ± 12.08. BMI of the participants varied from 12 to 36 kg/cm^2^ with the mean ± SD of 23.41 ± 4.33.

Regular SLT product use in the form of* gutka*,* khaini*,* pan masala* with tobacco, or tooth powder containing tobacco was elicited in 14.4% of the participants.

### 3.1. Statistical Analysis

A positive correlation was observed between blood pressure and age, the quantity of SLT consumed, and BMI as depicted in Table 1. The correlation coefficient for each of the above parameters was found to be statistically significant (*P* < 0.001) except the correlation between diastolic blood pressure and quantity of SLT consumed (Table 1). The regression equation shows that a unit change in age brings an average change of 0.74 times in systolic blood pressure (Table 1). Similarly, a unit change in the quantity of SLT consumed and BMI raises the systolic blood pressure by 0.52 and 1.22 times, respectively (Table 1). For diastolic blood pressure, a unit change in age and BMI raises the diastolic blood pressure by 0.16 and 0.76, respectively (Table 1).

Multiple linear regression analysis showed that high systolic blood pressure was associated with age, high BMI, low level of education, and quantity of SLT with *P* value < 0.05 for each of the variables (Table 2). On the other hand, high diastolic blood pressure was associated only with high BMI and low level of education (Table 2). Age played no role in determining diastolic blood pressure. The monthly income which is often used as a marker of socioeconomic status played no role in determining the systolic and diastolic blood pressure.

## 4. Discussion

Hypertension (HTN) is one of the most important noncommunicable disease attaining epidemic proportions across the world as reported by WHO in 2013. HTN is one of the most important contributors to coronary artery disease, cerebrovascular diseases, premature deaths, and disability [1]. An estimated 54% of stroke cases and 47% of ischemic heart disease globally have been attributed to HTN [13]. The updated JNC-8 guideline recommendations for HTN classify risk factors as nonmodifiable (age, race, and family history) and modifiable. The latter include overweight and obesity, lack of physical activity, tobacco and excessive alcohol consumption, high sodium diet, stress, diabetes, and sleep apnea [3].

Overweight and obesity have been identified as a significant and independent risk factor for HTN and its associated cardiovascular complications [14]. The SEEK study reported the prevalence of obesity by conventional standards in 17.9% of hypertensive subjects [15]. The prevalence was much higher in the KEEP study with 78.6% of obese participants having HTN [16]. Bansal et al., in their study of a rural Indian community, showed that BMI was an independent predictor of HTN. The risk for development of HTN was higher among females than among males [5]. The present study, including only nonpregnant females, revealed a significant correlation of BMI with systolic (*P* < 0.001) as well as diastolic blood pressure (*P* < 0.001). This association assumes significance since weight is a modifiable risk factor for HTN and its complications.

Age has been documented as a vital nonmodifiable risk factor for HTN and cardiovascular diseases in numerous studies [5, 15]. Our study gave similar results with a positive correlation with systolic (*P* < 0.001) as well diastolic (*P* = 0.019) blood pressure.

Tobacco consumption, both as smoking and in smokeless form, has been associated with adverse cardiovascular effects. Smoking has been shown to be a risk factor for coronary heart disease and ischemic stroke [17]. Nicotine, the main constituent, is capable of activating the sympathetic nervous system, thereby increasing systolic blood pressure [18]. However, several studies have not found any significant increase in blood pressure levels among cigarette smokers compared to nonsmokers [6, 19]. Other studies have shown some association of HTN with smoking in a dose-response manner but not with current smoking status [7].

Tobacco consumption in smokeless forms is very common in India, especially among females [20]. Association of smokeless tobacco (SLT) consumption with adverse cardiovascular effects like ischemic heart disease and stroke has been reported in some studies [21, 22]. The literature search yielded conflicting results on an association of SLT use with HTN. Few Swedish studies have shown a higher prevalence of HTN among snuff users compared to nontobacco users [8, 23] while others have failed to detect such an association [9]. Studies from India, a region with high prevalence of chewing tobacco consumption, have shown a significantly increased prevalence of HTN in adult SLT users [10, 11]. Our study also revealed similar results with significant correlation of SLT use with an elevation of systolic (*P* = 0.003) but not with diastolic blood pressure (*P* = 0.16). A contributing factor, apart from nicotine, may be the high content of sodium as part of alkaline buffer required to facilitate nicotine absorption and the quantity of licorice in the flavor [24]. Of note is the fact that while earlier Indian studies had a predominance of males in their study subjects, the present study included only nonpregnant females to assess the predictors of HTN in this subgroup.

One of the limitations of the present study was the inability to confirm SLT usage with biochemical analysis. The history of SLT use, as well as the amount consumed, was based on self-assessment. However, an attempt was undertaken to avoid reporting bias on these aspects by eliciting a detailed history of the type of SLT used, the packaging along with weight, and the average number of packs consumed per day.

The association of smoking with hypertension is well established in literature. However, the same is not true for smokeless tobacco where conflicting results have been reported in various studies. Hence, the present study was undertaken to lend some clarification to this controversy. In conclusion, the present study reaffirms the association of increasing age and body mass index with a higher prevalence of hypertension in female subjects. More importantly, long-term consumption of smokeless tobacco was seen to be associated with systolic hypertension. These results underscore the importance of educating hypertensive patients and their family regarding beneficial effects of cessation of smokeless tobacco in addition to diet and lifestyle modifications to control body mass index. A number of price and nonprice tobacco control measures have been undertaken by the government to tackle the tobacco menace. Price of smokeless tobacco products has gone up significantly due to increase in taxes. However, the effectiveness of this price increase in reducing the affordability of these products is still not amply clear. Direct and indirect advertising of smokeless tobacco products have been banned under the COTPA (Cigarettes and Other Tobacco Products Act) of 2003. Depiction of use of smokeless tobacco products has been prohibited in the content on television and movies. If such depiction is deemed necessary, a “Statutory Warning” has to be displayed during such scenes for clearance by the requisite censor board [25]. The manufacture, sale, transportation, and storage of smokeless tobacco products have also been restricted by most of the states in India [26]. As a result of these measures, there has been a reduction in the prevalence of smokeless tobacco use (21.4% in GATS-2, 2016-17 compared with 25.9% in GATS-1, 2009-10) [27, 28]. However, the decline in prevalence of SLT use and SLT related health conditions has not met the expectations of stakeholders due to alternate marketing strategies adopted by the tobacco companies such as deceptive advertising of nontobacco products with the same brand name, easy availability, and lower cost compared to cigarettes [26]. Moreover, there are many organizations, including the tobacco industry itself, which play a role in protecting the livelihood of tobacco harvesting farmers in India.

## Figures and Tables

**Table 1:** Correlation and linear regression SBP^1^ and DBP^2^.

Parameters (*N* = 319)	Blood pressure	*r*-value (*P* value)	Regression equation
Age versus	SBP	0.376 (0.000)	SBP = 98.6 + 0.74 × age
	DBP	0.131 (0.019)	DBP = 74.31 + 0.16 × age

Quantity of smokeless tobacco (packets per day × years of consumption) versus	SBP	0.199 (0.000)	SBP = 126.13 + 0.52 × quantity of smokeless tobacco (packets per day × years of consumption)
	DBP	0.102 (0.068)	DBP = 80.08 + 0.17 × quantity of smokeless tobacco (packets per day × years of consumption)

BMI^3^ versus	SBP	0.267 (0.000)	SBP = 98.6 + 1.22 × BMI
	DBP	0.273 (0.000)	DBP = 62.57 + 0.76 × BMI

(1) SBP = systolic blood pressure; (2) DBP = diastolic blood pressure; (3) BMI = body mass index.

**Table 2:** Predictors of SBP and DBP (multiple linear regression approach).

Variable	SBP		DBP	
	*β* (standard error)	*P* value	*β* (standard error)	*P* value
Age	0.635 (0.100)	0.000	0.104 (0.065)	0.111
Quantity of smokeless tobacco (packets per day × years of consumption)	0.389 (0.131)	0.003	0.122 (0.087)	0.160
BMI	1.185 (0.225)	0.000	0.766 (0.148)	0.000
Level of education	−1.236 (0.617)	0.046	−1.013 (0.394)	0.011

Systolic and diastolic blood pressure are adjusted with age, quantity, BMI, and level of education.
